# Dimethyl Sulfoxide and Sodium Chloride Modulate the Crystal Structure in PMIA to Enhance Dyeing Performance: Molecular Dynamics Simulation and Experimental Investigations

**DOI:** 10.1002/advs.202414544

**Published:** 2025-02-23

**Authors:** Yan Zhuo, Kuang Wang, Minghui Chen, Zhengke Fan, Zhuangzhuang Sun, Jianli Liu, Yizheng Fu, Aixue Dong, Bo Zhu

**Affiliations:** ^1^ College of Textile Science and Engineering Jiangnan University Wuxi 214122 China; ^2^ Shaanxi Yuanfeng Textile Technology Research Co., LTD Shaanxi 710038 China; ^3^ School of Materials Science and Engineering North University of China Taiyuan 030051 China; ^4^ Key Laboratory of Intelligent Textile and Flexible Interconnection of Zhejiang Province Zhejiang Sci‐Tech University Hangzhou Zhejiang 310018 China; ^5^ Shaoxing Sub‐center of National Engineering Research Center for Fiber‐based Composites Shaoxing University Shaoxing Zhejiang 312000 China

**Keywords:** crystal structure modulation, DMSO/NaCl, dyeing performance, meta‐aramid (PMIA), molecular dynamics simulation

## Abstract

The widespread use of meta‐aramid (PMIA) is limited by its poor dyeing performance, and researchers often struggle to qualitatively and quantitatively assess its microscopic structural regulation when attempting to improve dyeing performance. Herein, DMSO/NaCl is chosen to synergistically modulate the structure of PMIA to improve its dyeing properties in combination with experiments and simulations. Initially, characterization and color testing reveal that the DMSO/NaCl combination induces structural changes in the amorphous regions of the PMIA fiber, improving the dispersion of the dye solution. Notably, PMIA exhibited a significant improvement in dyeing performance, with the K/S value increasing from 2.6 to 16.0 and dye uptake rising from 20.4% to 73.2%, while maintaining excellent colorfastness and mechanical integrity. Molecular dynamics simulations further confirm that DMSO/NaCl disrupts the hydrogen bonding network in the amorphous regions of PMIA, enhancing the mobility of molecular chains and increasing the free volume, thus providing additional adsorption and binding sites for the dye molecules. These findings highlight the potential of combining experimental and computational approaches to optimize the structural regulation and dyeing performance of PMIA fibers.

## Graphical abstract

This study combines experiments and molecular dynamics simulations to investigate the effects of DMSO/NaCl on the microstructural modulation and dyeing properties of meta‐aramid (PMIA). The findings suggested the effect of DMSO/NaCl disrupts the hydrogen‐bonding network of PMIA, improves the movement of molecular chains, increases the free volume of PMIA, and significantly improves the dyeing properties of PMIA fibers.

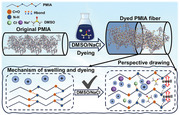

## 1 Introduction

Meta‐aramid,^[^ ^1^, ^2^, ^3^ ^]^ also referred to as Poly‐m‐phenylene isophthalamide (PMIA), is widely used, e.g., in aerospace, protective clothing, and electronic insulation materials due to its exceptional heat resistance, chemical stability, and mechanical strength.^[^ ^4^, ^5^, ^6^, ^7^, ^8^ ^]^ The demands for fabric color have correspondingly increased following the application fields of PMIA continue to expand and diversify, bringing the dyeing performance of PMIA into focus.^[^ ^9^ ^]^ However, PMIA faces significant challenges in dyeing due to its unique molecular structure and high crystallinity, which impede dye penetration and bonding, resulting in a complex dyeing process and suboptimal color outcomes. This limitation not only hinders the broader adoption of PMIA in the textile industry but also restricts its use in high‐end markets, where precise color customization and aesthetic requirements are critical. Therefore, investigating the effects of dimethyl sulfoxide (DMSO) and sodium chloride (NaCl) on the microstructure and dyeing performance of PMIA during the dyeing process is essential.

Previous studies have focused on the structural modulation of PMIA to improve its dyeing performance, with particular emphasis on carrier dyeing technologies.^[^ ^10^, ^11^, ^12^, ^13^ ^]^ Carrier dyeing involves the use of organic solvents or additives to penetrate PMIA fibers, weaken hydrogen bonds, and induce fiber swelling, thereby creating additional space and binding sites for dyes, which results in uniform dyeing and high colorfastness. However, the carriers employed often have unpleasant odors and toxicity,^[^ ^14^, ^15^ ^]^ raising environmental and safety concerns. In contrast, DMSO is a low‐toxicity, cost‐effective organic solvent with excellent solubility and effective PMIA structure modulation, making it a common PMIA spinning solvent and a material for PMIA paper production.^[^ ^16^, ^17^ ^]^ For instance, Tan et al. used a DMSO/KOH mixture to swell and dissolve aramid paper, which was then regenerated to modify its structure.^[^ ^18^ ^]^ Regarding dyeing performance, Moore et al. treated aramid fibers with solvents such as DMSO to disrupt polymer chain orientation and facilitate the formation of air voids, which improved the dyeing properties of PMIA when dyed with cationic dyes.^[^ ^19^ ^]^ However, the mechanisms through which DMSO regulates the PMIA structure and dyeing process were not thoroughly investigated. Furthermore, DMSO alone has limited effectiveness in enhancing PMIA dyeing performance. Studies also suggest that electrolytes, such as NaCl, can reduce the electrostatic repulsion between dyes and aramid fibers during the dyeing process, thereby promoting dye adsorption and diffusion within the fibers.^[^ ^20^ ^]^ Therefore, this study investigates a DMSO/NaCl system to synergistically modulate PMIA structure and enhance its dyeing performance.

The structure and dyeing properties of PMIA may exhibit opposing effects at different DMSO/NaCl concentrations. For instance, a low concentration of DMSO may not be sufficient to fully dissolve or swell the PMIA chains, resulting in strong interactions between PMIA molecular chains remaining, which limits the creation of sufficient space and binding sites for effective dye adsorption and penetration. In contrast, at high concentrations of DMSO, PMIA fibers may completely dissolve, which, while increasing the mobility of the molecular chains, could also compromise the mechanical properties of the material due to excessive dissociation of the PMIA chains.^[^ ^18^, ^21^ ^]^ Additionally, the concentration of NaCl can influence PMIA dyeing differently. Moderate concentrations of NaCl can reduce charge repulsion between dye molecules and the PMIA fabric, improving the dispersion of dye molecules.^[^ ^22^ ^]^ However, at high NaCl concentrations, dye molecules may aggregate excessively, reducing the stability of the dye solution. Therefore, it is necessary to investigate the microstructural effects of different DMSO/NaCl contents on PMIA to improve the dyeing properties of PMIA.

Traditional characterization techniques commonly used in materials science and chemical analysis, such as Fourier transform infrared spectroscopy (FT‐IR) and X‐ray diffraction (XRD), provide average structural or macroscopic property data. However, these methods have limitations in elucidating the microscopic mechanisms of PMIA structural regulation, such as the number of hydrogen bonds and macromolecular chain mobility, and fail to reveal changes at the nanoscale or microscale. The rapid advancement of computer science and theoretical chemistry has led to the widespread application of molecular dynamics simulations across various fields, including materials science, chemistry, biomedicine, and environmental science.^[^ ^23^, ^24^, ^25^ ^]^ For instance, Lyu et al. employed molecular dynamics simulations to calculate the radial distribution function and the variation in the number of hydrogen bonds in the ANF‐PVA organohydrogel system at different DMSO concentrations, reflecting improvements in mechanical properties.^[^ ^26^ ^]^ Sun et al. used molecular dynamics simulations to calculate the number of hydrogen bonds in a multiscale PMIA paper composed of both microscale and nanoscale PMIA fibers.^[^ ^27^ ^]^ Their results indicated that the fibers were able to form in situ hydrogen bonds at the interface, thereby contributing to the mechanical reinforcement of the multiscale PMIA paper.

In this study, the effect of different DMSO/NaCl contents on the structure and staining properties of PMIA was investigated by experimental and theoretical calculations cross‐validation. Initially, scanning electron microscopy (SEM), energy dispersive spectrometry (EDS), X‐ray photoelectron spectroscopy (XPS), FT‐IR, and XRD are used to characterize the surface morphology and structure of PMIA fibers before and after dyeing. The impact of DMSO on the dyeing performance of PMIA fibers is studied through K/S values, dye uptake rates, dye penetration, and colorfastness tests. Zeta potential and particle size measurements of the dye solution under different DMSO/NaCl contents analyze the effect of the system on the dye solution, while the tensile tests assess the mechanical properties of PMIA. Subsequently, structural models of PMIA with varying DMSO/NaCl contents are constructed in Material Studio. After geometric optimization and annealing for energy convergence, molecular dynamics (MD) simulations are conducted. The simulations calculate microscopic structural changes in PMIA, such as hydrogen bond quantity, radial distribution function (RDF), mean square displacement (MSD), free volume (FFV), and cohesive energy (CED).

## 2 Experimental Section

### 2.1 Experimental Materials

**Figure 1:**
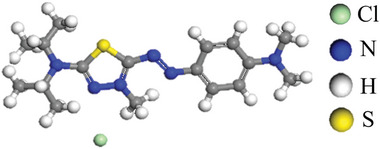
Molecular structure formula of cationic dye B‐159.

100% PMIA fabric (160 g m^−2^, twill weave, and linear density of 1.66 dtex) was obtained from Yantai Tayho Advanced Materials Group Co., Ltd., China. Cationic blue B‐159 (**Figure** **1**) was supplied by Wuxi Evan New Materials Technology Co., Ltd., China, and used without further purification. DMSO, NaCl, acetic acid, and anhydrous sodium acetate were purchased from Sinopharm Chemical Reagent Co., Ltd, China (analytical grade).

### 2.2 Dyeing Procedure

The dye solution was prepared with varying DMSO/NaCl concentrations under the following dyeing conditions: dye concentration 3 owf% (90 mg), bath ratio 1:30, pH 3–4, DMSO X wt.%, and NaCl Y g/L. Based on preliminary experiments, 50 wt.% DMSO and 40 g L^−1^ NaCl were selected as benchmarks.^[^ ^28^ ^]^ DMSO and NaCl contents and sample names are listed in **Table** **1**. The PMIA samples were heated at 276.15 K min^−1^ to 393.15 K and dyed for 60 min, with the temperature (393.15 K) and pH (3‐4) tightly controlled throughout the process. After dyeing, samples were washed and dried at 353.15 K for 30 min. Each dyeing test was repeated 10 times.

**Table 1:** The content of DMSO, and NaCl in the samples with the corresponding surrogate.

DMSO‐X% NaCl‐40 g L^−1^		DMSO‐50% NaCl‐Y g L^−1^	
DMSO	NUM	NaCl	NUM
0%	D‐0%	0g/L	N‐0g/L
10%	D‐10%	10g/L	N‐10g/L
20%	D‐20%	20g/L	N‐20g/L
30%	D‐30%	30g/L	N‐30g/L
40%	D‐40%	40g/L	N‐40g/L
50%	D‐50%	50g/L	N‐50g/L
60%	D‐60%	60g/L	N‐60g/L

### 2.3 Characterization

The surface morphology of dyed PMIA fabrics was captured with scanning electron microscopy (SEM, SU1510, Hitachi, Japan) operating at 5 kV under 5000 magnifications. The elemental composition variation on the dyed PMIA fabrics was determined using energy‐dispersive spectrometry (EDS). The surface chemistry and elemental composition of the dyed PMIA fabrics were further analyzed by X‐ray Photoelectron Spectroscopy (XPS K‐Alpha, Thermo Scientific, USA). Fourier Transform Infrared Spectrometer (FT‐IR, Nicolet, Thermo Fisher Scientific, USA) was used to test and analyze the composition and structure of dyed PMIA fabrics. The range of attenuated total reflection mode (ATR) was set at 500–4000 cm^⁻¹^ with a resolution of 1.0 cm^⁻¹^ and with an average of 64 scans. The crystallinity of PMIA fiber was tested using an X‐ray diffractometer (XRD, D2 PHASER A26‐X1‐A2E0B2A0, Bruker AXS, Germany) at voltage 40 V, testing current 30 mA, testing angle 5–50° and scan speed 5°/min.

The K/S value (color strength) of the dyed PMIA fabrics, i.e., the shade of color on the surface of the fabric, was measured at the wavelength of maximum dye absorption (610 nm) with a reflectance spectrophotometer (Datacolor 650, Datacolor, USA) under D65 illuminant at 10° standard. The Dye uptake was tested following the GB/T 23976.1‐2009 standard, employing a UV spectrophotometer (TU‐1900, Beijing Puxi General Instrument Co., Ltd., Beijing, China) to measure the absorbance of the dye solution at its maximum absorption wavelength. The dye uptake was then calculated using Equation 1, where E represents the dye uptake, *A*_1_ denotes the absorbance of the dye after dyeing and dilution by a factor of m, and *A*_0_ indicates the absorbance of the dye liquor before dyeing and dilution by a factor of *n*.

(1) $$E\left(\%\right)=\left(\frac{mA_{0}-nA_{1}}{mA_{0}}\right)\times 100\%$$

Level‐dyeing property was obtained by measuring the K/S values at 10 random points on each sample according to Equations (2) and (3).

(2) $$A=\frac{\sum_{i=1}^{n}{\left(K/S\right)}_{i}}{n}$$

(3) $$Sr=\sqrt{\frac{\sum_{i=1}^{n}{\left[{\left(K/S\right)}_{i}-A\right]}^{2}}{n-1}}$$

where (K/S)_i_ is the K/S value for each random point and Sr is the standard deviation of the K/S value, a lower Sr means better leveling. Colorfastness of PMIA fabric, including rub fastness, wash fastness, and resistance to sunlight fastness, was tested respectively according to GB/T 3920‐2008, GB/T 3921‐2008 and GB/T8427‐2008. Measurement of the size and Zeta potential of aggregates in different dye solutions using a Zeta particle size analyzer (Brookhaven, USA). Tensile tests of PMIA fabric were performed by a universal tensile tester (WDW‐1, Shengda Instrument, China) according to GB/T 3923. 1–2013, the fabric was cut into a 350 mm × 60 mm specimen and the raw edges were removed from both sides so that the specimen size was 350 mm × 50 mm. 12 measurements were made and the average value was taken to obtain the final strength value of the specimen.

### 2.4 Molecular Dynamics Simulation

**Figure 2:**
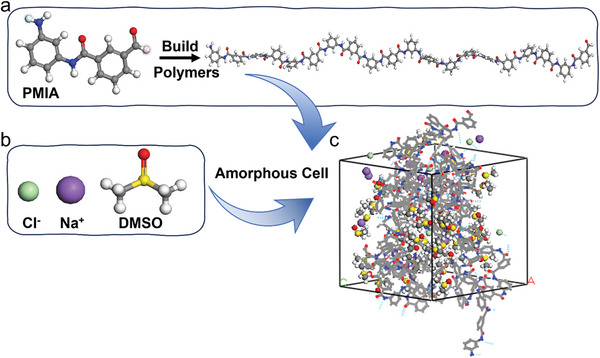
Modeling process of PMIA amorphous region under DMSO/NaCl solution system.

In this study, the amorphous region of PMIA was the main object of investigation Since the carrier and dye mainly act on the amorphous region of PMIA.^[^ ^28^ ^]^ In the Material Studio (MS) software,^[^ ^29^ ^]^ the macromolecular chains of PMIA, DMSO molecules, and Na^+^, Cl^−^ ion models were initially constructed. The molecular chain model (Polymerization degree of 10) was created using the Build Homopolymer module from PMIA monomers (**Figure** **2**a). Subsequently, force fields and charges were assigned to the constructed molecular models, followed by geometric optimization to minimize model energy. The Amorphous Cell module was used to construct PMIA structural models under various DMSO/NaCl contents (Figure 2c) with periodic boundary conditions. Model specific information is provided in Tables S1 and S2 (Supporting Information). Due to significant energy and structural deviations in the initial models, structural optimization was necessary to achieve minimized energy and stable configurations.

The model was first geometrically optimized with a mass of Medium and a maximum number of iteration steps of 2000. Subsequently, the Anneal module was applied to anneal the system with an annealing cycle of 8, an initial temperature of 300 K, a mid‐cycle temperature of 900 K for each annealing cycle, and a heating ramps per cycle of 10. The models were then optimized again, and the dynamic calculations were performed after the model had sufficiently converged. Since the dyeing temperature was 393.15 K, molecular dynamics simulations were conducted in the NPT (constant pressure and temperature) ensemble for 400 ps at 393.15 K to achieve reasonable density and structure values, followed by 200 ps of NVT (Constant temperature, volume, and number of particles) ensemble simulation. During the molecular dynamics simulations, the time step was set to 1 fs, with dynamic information collected every 5000 steps. All dynamic calculations were performed using the COMPASS III force field, which supports high‐precision polymer calculations and was applicable to both organic and inorganic molecules, with van der waals forces and electrostatic interactions calculated using the Atom and Ewald methods. Temperature and pressure were controlled using the Nose and Berendsen methods in the Forcite module. The original PMIA model equilibrium determination with densities is in Figure S1 (Supporting Information).

### 2.5 Model Calculation Characterization

In this study, geometric criteria were used to define hydrogen bonds in molecular dynamics simulations: the maximum distance between the acceptor and hydrogen atoms was set at 3.4 Å, and the minimum angle β was set at 90 degrees.^[^ ^30^, ^31^, ^32^ ^]^ In addition, hydrogen bonds (Length ≤ 2.5 Å) were considered to be strong because in this range, hydrogen bonds have higher bonding energies and exhibit stronger interactions. Hydrogen bonds (2.5 Å < Length ≤ 3.4 Å) were then defined as weak hydrogen bonds because the bonding energy was reduced and the strength of the interaction was weakened. Subsequently, the change in the number of hydrogen bonds of PMIA in the model was calculated and the RDF of H (N─H) and O (C═O) in the amide group of PMIA was counted.^[^ ^33^, ^34^ ^]^ Hydrogen bonding is defined in Figure S2 (Supporting Information). Equation (4) shows how to calculate RDF.

(4) $$g\left(r\right)=\frac{n_{B}V}{N_{B}4\pi r^{2}\Delta r}$$

where *n*_B_ denotes the number of B atoms at distance r from atom A in a shell layer of thickness *Δ*r, *V* means the space volume, and *N*_B_ is the total number of B atoms in the system.

MSD describes the deviation of a position of particles from its reference position after moving over time and is defined by Equation (5).^[^ ^35^, ^36^ ^]^ The coefficient of diffusion (D) is calculated by fitting the MSD curve Equation (6). In molecular dynamics simulations, MSD and the D were commonly used to study molecular diffusion behavior and the dynamic properties of the system. Thus, in MS, the Forcite analysis module is used to calculate the MSD and D of the PMIA polymer chain.

(5) $$MSD=\langle {\left|\vec{r_{i}}\left(t\right)-\vec{r_{i}}\left(0\right)\right|}^{2}\rangle$$

(6) $$D=\lim_{t\to \infty }\frac{\langle {\left|\vec{r_{i}}\left(t\right)-\vec{r_{i}}\left(0\right)\right|}^{2}\rangle }{6t}$$

where *r*_i_(0) and *r*_i_(t) are the vector displacements of any atom i in the system at the initial and t moments.

The size and distribution pattern of the free volume inside PMIA determines whether the dye molecules can diffuse smoothly into the fiber from a microscopic point of view. The changes in the FFV of the amorphous regions in PMIA were evaluated to elucidate the microstructural effects of DMSO/NaCl, which provide insights into the improved dyeing performance. FFV Calculated by Equation (7).

(7) $$FFV\left(r\right)=\frac{V_{f}}{V_{r}+V_{f}}\times 100\%$$

where *V*_f_ is the free volume of the system and *V*_r_ is the occupied volume of the system.

CED and solubility parameter (δ) were key indicators for measuring the compatibility of solvents with polymers, reflecting the strength of intermolecular interactions within the polymer.^[^ ^37^, ^38^ ^]^ The mechanism of how varying DMSO/NaCl concentrations affect PMIA during the dyeing process can be investigated by calculating the CED and solubility parameters of PMIA. CED and solubility parameters (δ) are calculated by Equation (8).

(8) $$\delta =\sqrt{CED}=\sqrt{\frac{\Delta E}{V}}=\sqrt{\frac{\Delta H_{m}-RT}{V}}$$

where ΔE is the CED of the system, *V* is the molar volume of the system, Δ*H*_m_ is the molar enthalpy of evaporation, R is the constant of the ideal gas (8.314·Jmol^−1^·K^−1^), and T is the absolute temperature.

## 3 Results and Discussion

### 3.1 Characterization and Performance Testing of Dyed PMIA

#### 3.1.1 Surface Morphology and Chemical Structure of PMIA

**Figure 3:**
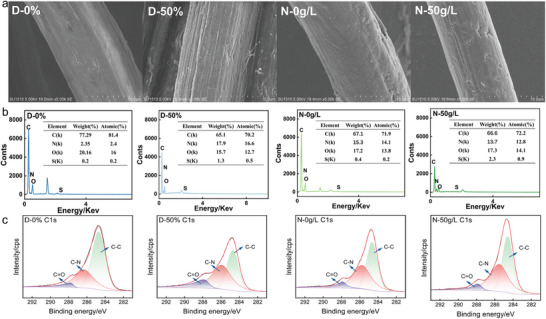
a) Surface morphology of dyed PMIA fibers. b) Elemental content of PMA fiber surface. c) Curve‐fitted C1s of PMIA fibers.

**Figure** **3**a shows the SEM images of the dyed PMIA surface at different DMSO and NaCl concentrations, while additional images of stained PMIA fibers are presented in Figure S4 (Supporting Information). The PMIA fibers in the D‐0% group, which did not contain DMSO, exhibit relatively smooth surfaces. The observed surface roughness and cracks are attributed to the spinning process of PMIA, where high‐temperature stretching during fiber formation induces the development of grooves and cracks on the fiber surface. In contrast, PMIA fibers in the D‐50% group showed more grooves, cracks, and higher surface roughness. This change suggests that the DMSO/NaCl treatment may have led to fiber swelling, which exacerbated the expansion of pre‐existing defects on the surface (e.g., cracks and grooves) and increased the surface roughness. The further increase in surface grooves and cracks on the N‐50 g L^−1^ PMIA fibers compared to N‐0 g L^−1^ suggests that the addition of NaCl further affects the morphological changes on the fiber surface on top of DMSO swelling. The possible reason for this is the influence of NaCl on the fiber structure by changing the solution environment or ionic effects, leading to a more significant increase in surface roughness.

The EDS spectra and corresponding elemental content tables for the D‐0%, D‐50%, N‐0 g L^−1^, and N‐50 g L^−1^ conditions are presented in Figure 3b. At the D‐0% condition, the surface elements of PMIA fibers are predominantly carbon (C‐77.29%) and oxygen (O‐20.16%), reflecting the large number of carbon chains and oxygen atoms in the PMIA structure. In the D‐50% group, the elemental content of C and O decreases, while the content of nitrogen (N) and sulfur (S) increases significantly, indicating enhanced diffusion and adsorption of N and S elements from the cationic dye B‐159 onto the surface of PMIA fibers. In the N‐0 g L^−1^ group, the addition of DMSO alone leads to a notable increase in nitrogen content, likely due to DMSO disrupting the hydrogen bond network within PMIA, resulting in fiber swelling and exposing more terminal groups on the surface.^[^ ^39^ ^]^ Meanwhile, in the N‐50 g L^−1^ group, the sulfur content on the PMIA surface increases markedly, suggesting that the synergistic effect of DMSO and NaCl facilitates more extensive dye adsorption.

Compared to the micron‐scale detection depth of EDS, XPS typically has a shallower detection depth of 4–10 nm for organic materials and polymers. Consequently, XPS provides information on the elemental distribution and content within just a few nanometers of the fiber surface. According to the result depicted in **Table** **2**, the N/C and O/C ratios for the other three groups increase to 0.10, 0.95, and 0.11, and decrease to 0.23, 0.22, and 0.23, respectively, compared to the N/C and O/C ratios of D‐0% group (0.08 and 0.25). This demonstrates that the surface of the fiber has more dyes adhering to it resulting in the masking of the O element. Given that the C element in B‐159 and PMIA exists in three different chemical environments, including the C─N and C─C groups present in both B‐159 and PMIA, and the C═O group in the PMIA amide bonds, it is necessary to perform peak fitting on the sub‐spectrum of C (Figure 3c). From the diagram, it can be seen that the proportion of C─C groups on the surface of the other three groups of fibers is less than that of D‐0%, and the C─N groups are more than that of D‐0%. This indicates that the C─N groups, which are more abundant in the dye structure, are more exposed on the fiber surface of the other three groups, suggesting that the synergistic effect of DMSO and NaCl makes the molecular chain structure of PMIA more open thus helping it to bind with more dyes to promote the embedding of dye molecules. Furthermore, the proportion of C═O groups on the PMIA surface increased upon the addition of DMSO, signifying an increase in the C─N ratio as well as the involvement of C═O groups attached to it in the amide group. This finding suggests that the DMSO/NaCl treatment leads to an increase in terminal groups and exposed amide groups on the PMIA surface.^[^ ^20^ ^]^

**Table 2:** The C, N, O, and S compositions of the PMIA.

Element	Atomic (%)			
	D‐0%	D‐50%	N‐0g/L	N‐50g/L
C1s	74.04	74.25	75.6	74.85
N1s	6.48	7.42	7.23	7.67
O1s	19.25	17.81	16.68	16.85
S2p	0.22	0.51	0.48	0.64

#### 3.1.2 Effect of DMSO/NaCl on Hydrogen Bonding and Crystallinity of PMIA

**Figure 4:**
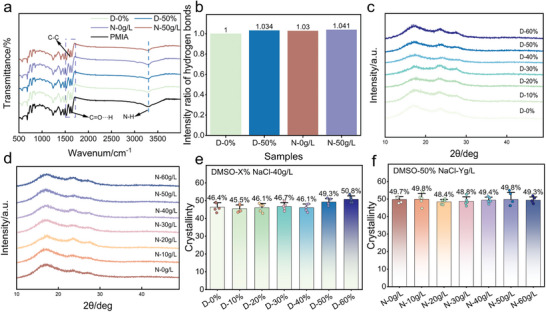
a) FT‐IR spectra. b) Intensity ratio of hydrogen bonds. c,d) XRD of different samples. e,f) Crystallinity of different samples.

One of the key factors influencing dye penetration is the hydrogen bonding interactions within the fiber structure during the dyeing process. As a strong hydrogen bond acceptor, DMSO can effectively weaken the hydrogen bonding forces within the fiber, increasing the molecular chain spacing and reducing fiber compactness, thereby providing more adsorption sites for the dye.^[^ ^40^, ^41^ ^]^ However, contrary to expectations, the N─H stretching vibration peak (3279 cm⁻¹) of the three groups of PMIA fibers—D‐50%, N‐0 g L^−1^, and N‐50 g L^−1^—shifted to lower wavenumbers, indicating a redshift (**Figure** **4**a), which signifies an enhancement of hydrogen bonding. The redshift reflects a decrease in vibrational frequency, suggesting that the increased intermolecular hydrogen bonding in PMIA after dyeing imposes greater constraints on the N‐H bond vibration. Additionally, changes in the relative strength of hydrogen bonds can be assessed by calculating the peak intensity ratio between the C═O···H stretching band at 1646 cm^−1^ and the aromatic ring C─C vibration band at 1606 cm^−1^ (Figure 4b).^[^ ^18^, ^42^ ^]^ The hydrogen bond strength increased to 1.034 at D‐0% and to 1.03 and 1.041 for samples at N‐0 g L^−1^ and N‐50 g L^−1^, respectively. The results of the redshift phenomenon and hydrogen bonding forces indicate that DMSO caused the rearrangement of the hydrogen bonding network of PMIA and enhanced the hydrogen bonding forces within the PMIA fibers.

The enhancement of hydrogen bonding contributes to the regular arrangement between molecular chains and thus promotes the increase of crystalline zones. Therefore, the crystalline and amorphous structures of PMIA were determined by XRD to investigate the changes and mechanisms of the crystalline and amorphous structures of PMIA after dyeing in the DMSO/NaCl system at different contents.

The XRD patterns of PMIA treated with different DMSO/NaCl concentrations (Figure 4c,d) show that the peak shape and position of the crystalline regions remain unchanged, indicating that DMSO/NaCl treatment does not disrupt the crystalline structure of PMIA. When the DMSO concentration increases to 50% and 60%, the XRD peaks of PMIA become sharper, and the crystallinity rises to 49.3% and 50.8% (Figure 4e), respectively. This observation aligns with the calculated hydrogen bonding strength, suggesting that only when the DMSO concentration reaches a certain level does the DMSO/NaCl treatment significantly enhance the hydrogen bonding and crystallinity of PMIA. This effect is likely due to the reorganization of molecular chains in the heated dyeing environment triggered by DMSO/NaCl, leading to the expansion of the crystalline regions and the strengthening of the hydrogen bond network. Additionally, the penetration of dye further fills the voids in the amorphous regions, resulting in increased hydrogen bonding strength and crystallinity of PMIA. When the DMSO concentration is fixed at 50%, changes in NaCl concentration have less effect on the crystalline structure of PMIA, with crystallinity fluctuating ≈49% (Figure 4f), but still higher than the initial 46.4% at D‐0%. This indicates that NaCl has a limited regulatory effect on the crystalline regions of PMIA. Overall, the regulation of hydrogen bonding and crystallinity in PMIA by DMSO/NaCl treatment is highly concentration‐dependent and is only significant at suitable DMSO concentrations.

#### 3.1.3 Dyeing Performance

**Figure 5:**
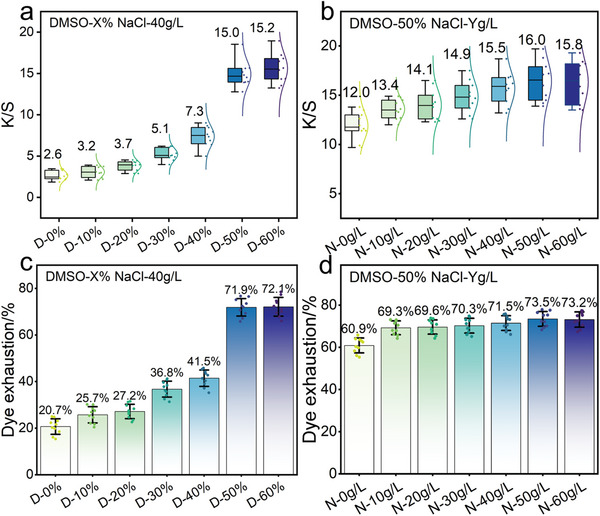
a,b) K/S values of different samples. c, d) Dye uptake of different samples.

The K/S value and uptake rate of dyed PMIA fabrics were tested to verify the prediction that DMSO/NaCl synergistically swells PMIA fibers and promotes dye penetration during dyeing. Whilst NaCl improves dyeing by reducing electrostatic repulsion between dye and fiber, D‐0% containing only NaCl had the lowest K/S value (2.6) with a dyeing rate (20.7%) (**Figure** **5**a, c). Afterward, the values of K/S and uptake rate slowly increased with the concentrations of DMSO to 7.3 and 41.5% at D‐40%, respectively, and significantly increased to 15.0 and 71.9% at D‐50%. More obvious enhancement of the dyeing performance of PMIA was achieved by the increase of NaCl content, with the K/S value and the dyeing rate increasing from 12.0 and 60.9% for N‐0 g L^−1^ to 16.0 and 73.5% for N‐50 g L^−1^ (Figure 5b,d). The results of PMIA dyeing enhancement showed that DMSO reached a certain threshold (e.g., 50%) in the DMSO/NaCl system to dissolve the PMIA fiber structure significantly. Meanwhile, NaCl could only have a better dyeing promotion effect in the fiber dissolution environment provided by DMSO. Moreover, the K/S value and the dyeing rate of PMIA at N‐60 g instead decreased to 15.8 and 73.2%, which may be attributed to the excessive ionic strength leading to the aggregation of dyes and triggering the contraction of the fiber structure.

**Figure 6:**
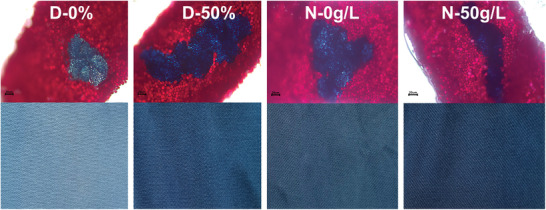
Cross‐section and surface photographs of dyed PMIA fabrics.

The standard deviations of the K/S values of dyed PMIA fabrics are shown in **Table** **3**. The relatively large standard deviation values (0.068, 0.042) of the dyed fabrics at D‐0% and N‐0 g L^−1^ indicate that the dyes are not evenly distributed on the fabric surface. The standard deviation values of the dyed fabrics under the conditions of D‐50% and N‐50 g L^−1^ were significantly reduced to 0.014 and 0.011, respectively, indicating that the leveling effect was significantly improved. Contrary to the uniform distribution of the dye in D‐50%, many fibers of PMIA in D‐0% were not penetrated by the dye, and obvious dye aggregation appeared (**Figure** **6**). In addition, the interface color of PMIA fibers with N‐50 g L^−1^ was uniform and the color difference was significantly reduced compared with that of the N‐0 g L^−1^ group, which indicated the obvious solubilizing effect and dye‐promoting effect of DMSO/NaCl on PMIA fibers. Additional dyed fabric photos are in Figure S5 (Support Information).

**Table 3:** Levelness of dyed PMIA fabric.

Levelness (standard deviation) Sr							
D‐X%	D‐0%	D‐10%	D‐20%	D‐30%	D‐40%	D‐50%	D‐60%
Sr	0.068	0.038	0.038	0.036	0.019	0.014	0.016
N‐Yg/L	N‐0g/L	N‐10g/L	N‐20g/L	N‐30g/L	N‐40g/L	N‐50g/L	N‐60g/L
Sr	0.042	0.033	0.013	0.022	0.013	0.011	0.014

#### 3.1.4 Colorfastness Performance

Colorfastness tests were conducted on different samples, and the overall results are summarized in **Table** **4**. At a DMSO concentration of 30 wt.%, the rub fastness decreased by half a grade, indicating that although the coloration of the fabric deepened, the bond between the fabric and the dye was not strong, with many dye particles floating on the surface. The increase in lightfastness is attributed to the additional surface dye, which protects the PMIA fibers and internal dye.^[^ ^43^, ^44^ ^]^ Both the D‐50% and N‐50 g L^−1^ groups showed comprehensive improvements in colorfastness, with the sunlight fastness of the N‐50 g L^−1^ group being half a grade higher than that of the D‐50% group. This revealed that the synergistic promotion of dyeing by DMSO and NaCl was better at 50 wt.% DMSO and 50 g L^−1^ NaCl, and deeper coloration of PMIA fabrics was achieved while maintaining better colorfastness.

**Table 4:** Effect of DMSO treatment on the Colorfastness of PMIA fabric.

	Rubbing fastness		Soap Wash fastness		Sunlight fastness
Sample	Dry	Wet	Color change	Staning	
PMIA	4	3‐4	4	4	1
D‐0%	4	3‐4	4	4	1
D‐10%	4	3‐4	4	4	1
D‐20%	4	3‐4	4	4	1
D‐30%	3‐4	3	4	4	1‐2
D‐40%	3‐4	3‐4	4	4	1‐2
D‐50%	4‐5	4	4‐5	4‐5	2‐3
D‐60%	4	3‐4	4	4	2
N‐0g/L	4	3	4	4	1‐2
N‐10g/L	4	3	4	4	1‐2
N‐20g/L	4	3	4	4	1‐2
N‐30g/L	4	3	4	4	1‐2
N‐40g/L	4‐5	3‐4	4	4	2‐3
N‐50g/L	4‐5	4	4‐5	4‐5	3
N‐60g/L	4‐5	3‐4	4‐5	4‐5	2‐3

#### 3.1.5 Zeta Potential and Particle Size

**Figure 7:**
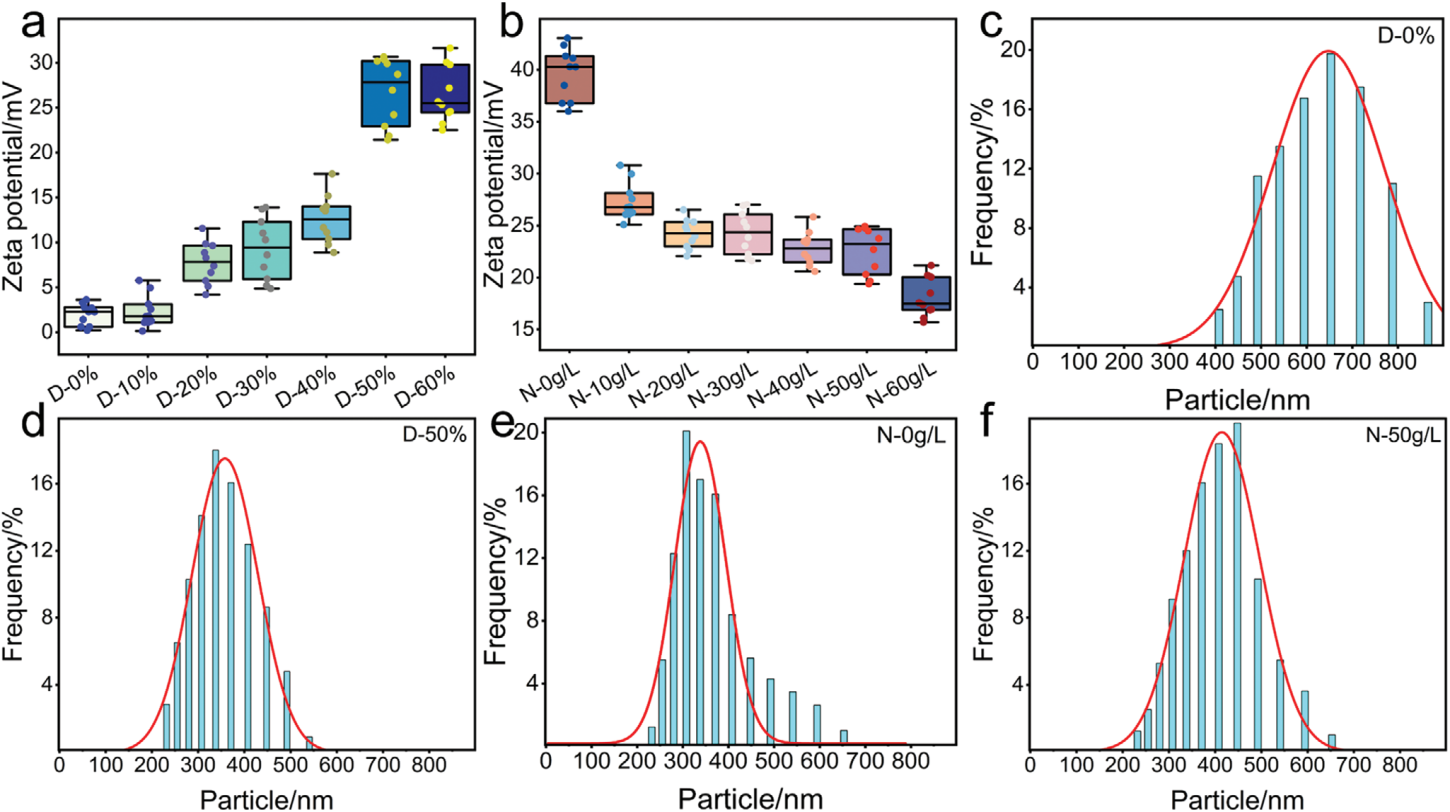
a, b) Zeta potential of different samples. c–f) Grain size distribution curve of (c) D‐0%; (d) D‐50%; (e) N‐0 g L^−1^; (f) N‐50 g L.

The zeta potential and particle size of the dye solution were tested at different DMSO/NaCl contents, to verify the effect of the DMSO/NaCl system on the aggregation behavior of the dye solution. The zeta potential of cationic dye stains at different DMSO concentrations (**Figure** **7**a) showed that the zeta potential of the stains increased gradually with the increase of DMSO concentration, peaking at D‐50% and D‐60%, where it remains relatively stable. This increase in zeta potential suggests that DMSO not only maintains the swelling effect on PMIA but also enhances the electrostatic repulsion between dye micelles, reducing dye aggregation in the solution.^[^ ^45^ ^]^ This prevention of excessive dye aggregation on the fiber surface or within ensures uniform dyeing. The zeta potential of the dye solution at different NaCl concentrations (Figure 7b) showed that the zeta potential of the dye solution gradually decreased with the increase of NaCl This indicates that NaCl can shield the charges on the dye molecules, compressing the electrical double layer and thus lowering the zeta potential.^[^ ^46^ ^]^ Consequently, the electrostatic repulsion between dye molecules is weakened, making them more prone to aggregation.

The particle sizes of D‐0%, D‐50%, N‐0 g L^−1^, and N‐50 g L^−1^ are shown in Figure 7c–f, respectively. Consistent with the zeta potential results, the dye particle size decreases from 660 nm(D‐0%) to 320 nm(D‐50%) as the DMSO concentration increases. This finding illustrates that the solvent action and polarity of DMSO disrupt the molecular forces between the dye aggregates, which may be related to the fact that the S═O group in DMSO can form a hydrogen bonding interaction with B‐159 and thus depolymerize the dye aggregates. Conversely, under N‐50 g L^−1^ conditions, the particle size increases (410 nm), surpassing that of D‐50% (320 nm) and N‐0 g L^−1^ (300 nm). It indicates that a high concentration of NaCl can reduce the potential of the dynamic layer of dye micelles in the dye solution, and shield the electrostatic repulsion between dye molecules to make the dye molecules more easily aggregated, which reduces the stability of the solution.

#### 3.1.6 Mechanism of DMSO/NaCl Promoting PMIA Dyeing

**Figure 8:**
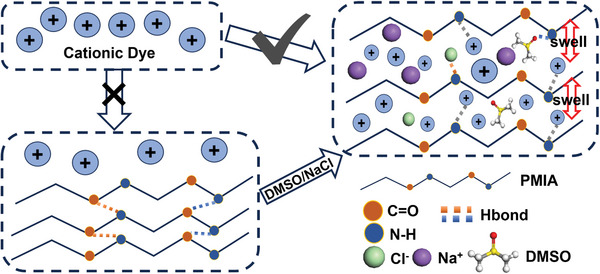
Schematic representation of DMSO/NaCl‐induced PMIA fiber structural modulations and enhanced dye adsorption pathways.

In conclusion, the dyeing mechanism of the DMSO/NaCl system can be summarized as follows: as shown in **Figure** **8**, DMSO disrupts or weakens the internal hydrogen bond network of PMIA fibers, loosening the fiber structure and providing more pathways and space for dye molecule penetration and adsorption. Meanwhile, the addition of Na^+^ and Cl^−^ ions neutralizes the negative charges on the fiber surface, reducing the electrostatic barriers between the dye and the fiber, thereby facilitating dye adsorption. As the NaCl concentration increases, the diffusion of dye molecules within the fiber accelerates, allowing for more uniform distribution on the fiber surface and deeper penetration, thus enhancing dyeing efficiency. Therefore, the synergistic effect of DMSO and NaCl significantly improves the dyeing performance of PMIA, though careful control of NaCl concentration is necessary to avoid potential negative effects such as dye aggregation and fiber shrinkage.

#### 3.1.7 Mechanical Properties of PMIA Fabrics

**Figure 9:**
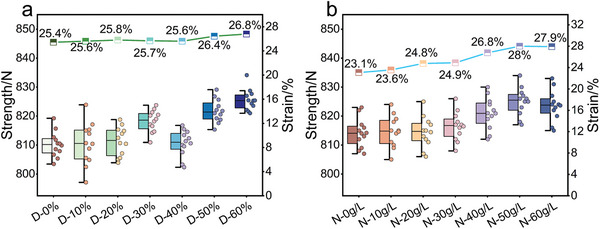
Breaking strength and elongation at break of different samples.

It is essential to maintain the mechanical properties of PMIA during the dyeing process because the excellent mechanical properties of PMIA are the basis for its wide range of industrial applications. The tensile strength and elongation at the break of dyed PMIA fabrics under different DMSO concentrations are presented in **Figure** **9**a. The tensile strength of PMIA fabrics fluctuated ≈810 N within the 0–40 wt.% DMSO concentration range. However, when the DMSO concentration reached 50 and 60 wt.%, the tensile strength of the PMIA fabrics increased to ≈825 N and 830 N, respectively. As depicted in Figure 9b, the increase in NaCl concentration similarly led to a gradual rise in the tensile strength of the dyed PMIA fabrics. At 0 g L^−1^ NaCl concentration, the tensile strength was ≈810 N. With further increases in NaCl concentration to 50 g L^−1^ and 60 g L^−1^, the tensile strength improved to ≈825 and 820 N, respectively. The mechanical performance tests reveal that the synergistic interaction of DMSO and NaCl regulates the fiber structure at the molecular level. DMSO swells the fibers, allowing the chain segments to move and align more freely, enhancing fiber extensibility. The role of NaCl may be due to the fact that Na⁺ and Cl‐ ions enhance the contact and bonding of the molecular chains by shielding the charges between the PMIA fiber molecules and attenuating the electrostatic repulsive forces between the negative charges. This effect helps to promote the re‐formation of hydrogen bonds between fiber molecules.^[^ ^47^ ^]^ In addition, Na⁺ and Cl‐ ions may also further stabilize the internal structure of the fiber molecules by binding to the charged groups in the fiber molecules, thus enhancing intermolecular interactions. The combined effect of DMSO and NaCl enables PMIA fibers to exhibit superior mechanical properties under stress.

### 3.2 Mechanism Analysis of the Microstructural Effect of DMSO/NaCl on PMIA

#### 3.2.1 Modulation of PMIA Hydrogen Bonding Network by DMSO/NaCl

The effects of different DMSO/NaCl contents on the hydrogen bonding network in the non‐crystalline region of PMIA were calculated based on molecular dynamics simulations to investigate the microstructural changes of PMIA during the dyeing process with DMSO/NaCl. The polar functional group of DMSO (S═O) will interact with the hydrogen bond donor N─H or acceptor C═O on the amide bond in PMIA when PMIA is stained under the DMSO/NaCl system, thus interfering with or substituting for the two types of hydrogen bonds, N‐H···O, N‐H···N, formed by the amide bond of the original PMIA(Figure S3, Supporting information).^[^ ^8^, ^48^ ^]^ At the same time, dissociating ions from NaCl (Na^+^ and Cl^−^) can destabilize the fiber structure by acting on the hydrogen bonding network of PMIA When the ionic environment around PMIA fibers is altered by NaCl.

**Figure 10:**
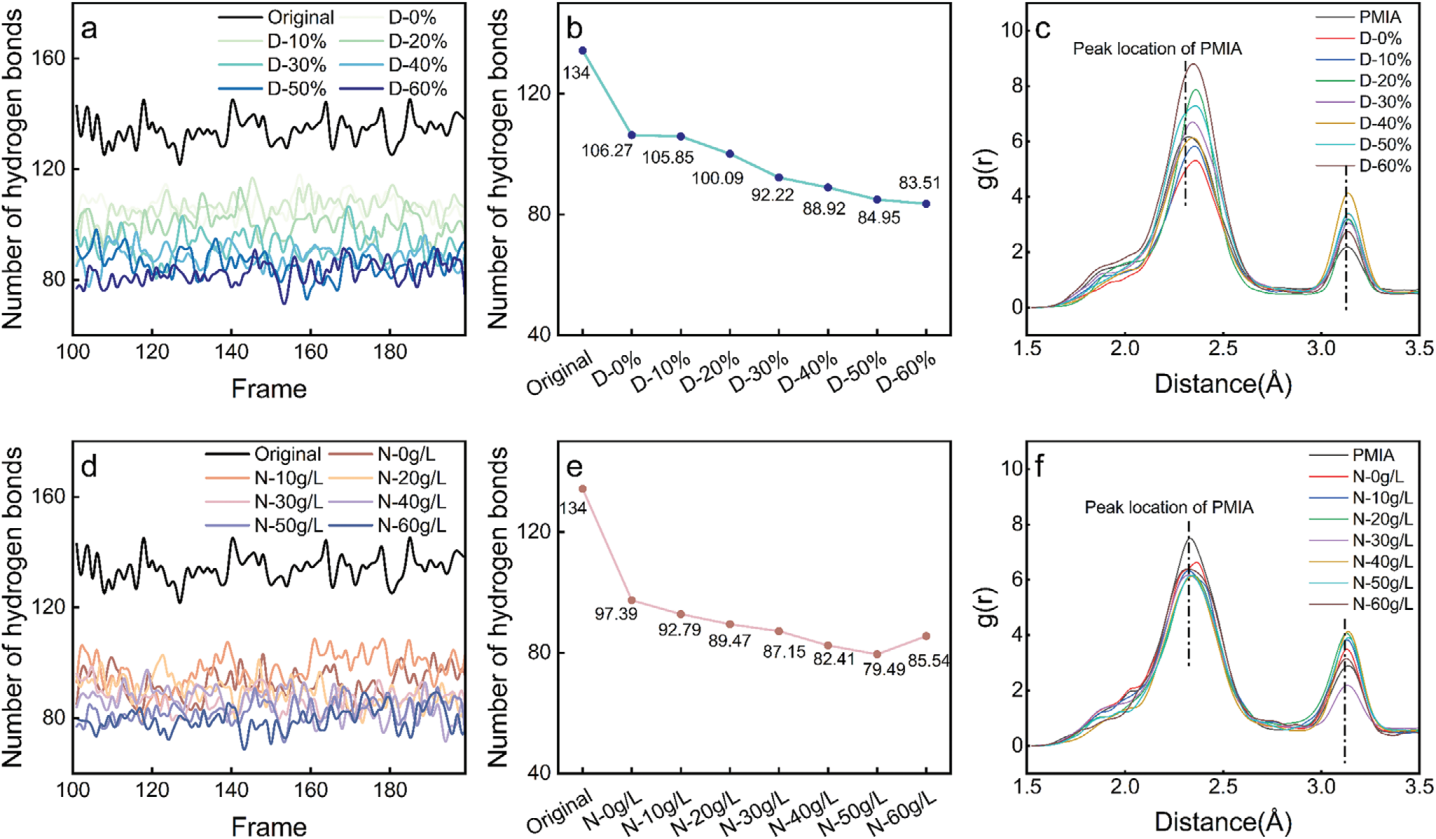
a, d) change in the number of hydrogen bonds. b, e) Average number of hydrogen bonds within PMIA. c, f) RDF curves for H(N─H) to O(O═C) in PMIA.

The number of hydrogen bonds in the non‐crystalline regions of PMIA fluctuates significantly over time are depicted in **Figure** **10**a, d. This result does not indicate instability in the model but rather reflects the dynamic nature of hydrogen bond formation and dissociation in the less orderly non‐crystalline regions, where molecular chains exhibit some degree of mobility. As illustrated in Figure 10b, the average number of hydrogen bonds in the non‐crystalline regions of PMIA decreases from an initial 134 to 106.27 at D‐0%, indicating that the dissociated ions from NaCl interfere with the hydrogen bond network of PMIA. The magnitude of the decrease in the number of PMIA hydrogen bonds becomes larger at D‐30%, which is consistent with the experimental significant increase in the K/S value of PMIA fabrics when DMSO reaches a certain threshold. At this stage, the interior of the fiber provides more binding sites for the adsorption and penetration of the dye molecules, thereby improving the dyeing performance of PMIA.

The average number of hydrogen bonds decreases from 134 in the initial state to 79.49 when the NaCl concentration reaches 50 g L^−1^ (Figure 10e), lower than the 83.51 observed at D‐60%. This indicates that the decline in the K/S value of PMIA at N‐60 g L^−1^ in the dyeing experiments is partly due to the excessive NaCl enhancing the interactions between fiber molecular chains, promoting the formation of additional hydrogen bonds within the fiber. This tighter molecular bonding reduces the pathways available for dye molecule penetration.

The RDF of H(N─H) to O(O═C) in PMIA was calculated to assess the probability and strength of hydrogen bond formation in DMSO/NaCl for PMIA. The RDF curve shows a first peak at 2.3 Å (Figure 10c,f), which corresponds to the typical hydrogen bond distance, indicating the formation of strong hydrogen bonding interactions between amide groups at this point, contributing to the stability of fibers. The peak at 3.2 Å, on the other hand, reflects weaker hydrogen bonding interactions, which can enhance the flexibility and dyeability of fibers, as dye molecules can more easily penetrate through these looser structures into the fiber.

In the RDF curves of PMIA at varying DMSO concentrations, a rightward shift in the peak position is observed (Figure 10c), suggesting an increased likelihood of longer hydrogen bond formation and, consequently, a weakening of hydrogen bonding interactions. However, the RDF peak at 2.3 Å deviates from expectations. Since the parameters 𝑁_𝐵_, 𝑟 and Δ𝑟 are the same for different PMIA models in the RDF calculation formula (Equation. 4), the molecular volume *V* increases with higher DMSO content. Therefore, *V* significantly influences the RDF calculation outcomes. The peaks of D‐0% and D‐10% are lower than those of pure PMIA, indicating a reduced probability of hydrogen bond formation, which aligns with the hydrogen bond count results. The above hydrogen bonding calculations showed a significant decrease in the number of hydrogen bonds at D‐50% and D‐60%, which is consistent with the expectation that DMSO acts as a strong hydrogen bonding acceptor interfering with hydrogen bonding within PMIA. However, the RDF calculations showed an increase in the peak of the RDF curves for both, which is the exact opposite of the hydrogen bonding calculations may be attributed to the fact that the high concentration of DMSO molecules significantly increased the total volume of the system, V, which led to an underestimation of the denominator value of the RDF, resulting in a high g(r). Notably, despite the larger 𝑉 value in the D‐40% system, its RDF peak at 2.3 Å remains unchanged compared to pure PMIA but shifts to the right. This indicates a reduced probability of hydrogen bond formation and an increase in hydrogen bond length, suggesting a weakening of the hydrogen bond strength between molecular chains. Additionally, the increased RDF peak at 3.1 Å in the D‐40% system signifies that the DMSO/NaCl system converts stronger, short hydrogen bonds within PMIA into weaker, longer hydrogen bonds, thereby facilitating dye penetration and adsorption.

The RDF calculation results for the PMIA models are minimally affected due to the relatively small variation in *V* values across different NaCl concentrations. This trend suggests that the probability of hydrogen bond formation initially diminishes and subsequently increases. Furthermore, the rightward shift of the peak position is followed by a leftward shift, indicating the formation of stronger, shorter hydrogen bonds, which aligns with the hydrogen bond count results. These findings demonstrate that the synergistic effect of DMSO and NaCl significantly modulates the hydrogen bond network within PMIA. As the DMSO content increases, the hydrogen bond network within PMIA weakens; however, a certain concentration of NaCl enhances the probability of hydrogen bond formation, promoting the reconstruction of the hydrogen bond network. This mechanism explains the retention of PMIA mechanical properties observed experimentally. In addition, the current model has been geometrically optimized and annealed to ensure its structural stability and reasonableness, so the inaccuracy of the RDF results does not stem from a flaw in the model construction process.

#### 3.2.2 Chain Motion of PMIA

**Figure 11:**
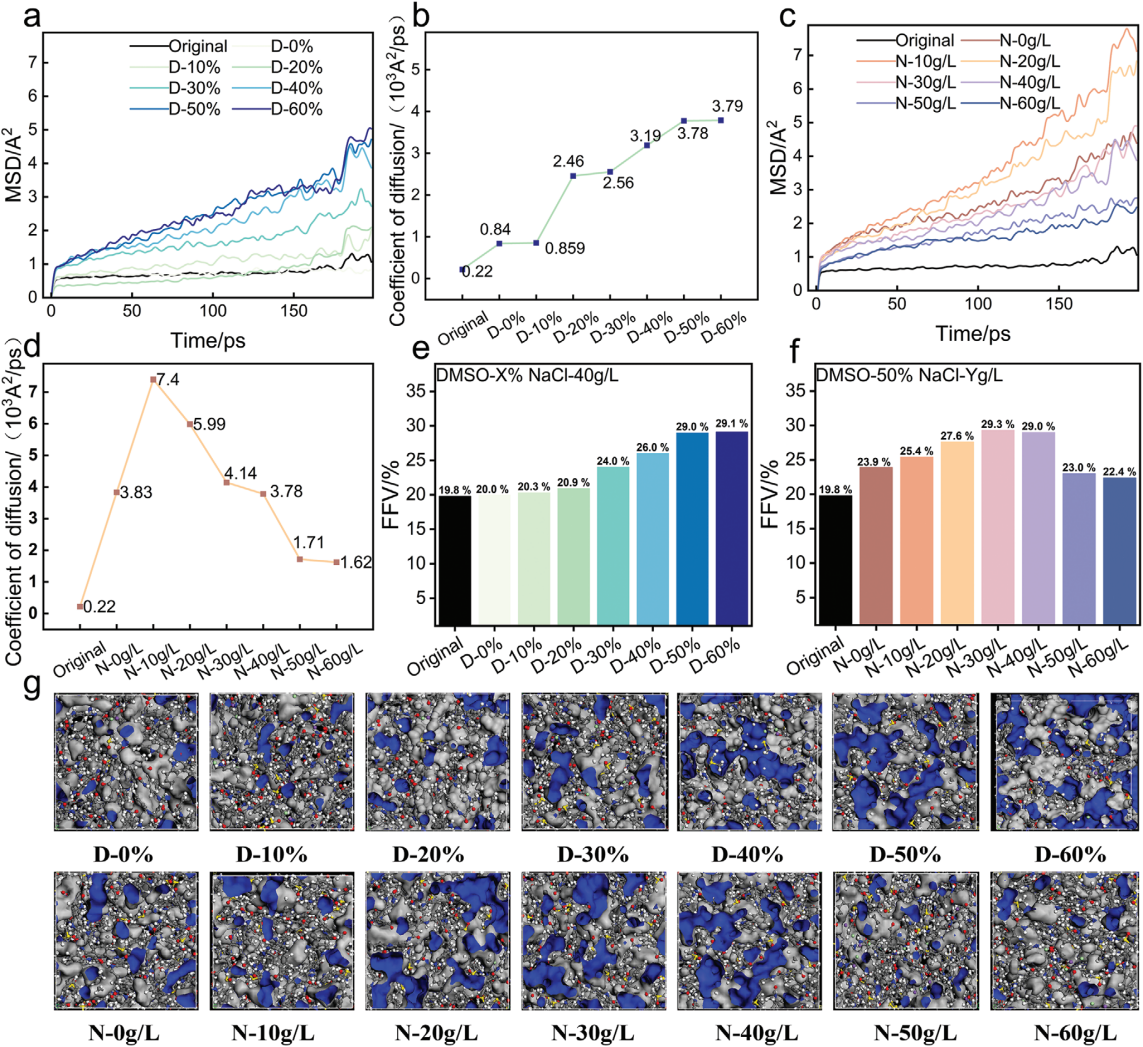
a, c) MSD values of different models. b, d) values of different models. e, f) FFV of different models. g) Schematic of the free volume of the model.

The MSD and D of PMIA in different DMSO contents are shown in **Figure** **11**a, b. The MSD and diffusion coefficients of the original PMIA were low, indicating that its molecular chains have poor kinematic properties. The MSD and D of PMIA exhibit a continuous increase with the addition of DMSO, showing a significant growth around D‐20%, and then stabilizing after D‐50%. This is consistent with the hydrogen bonding results, where DMSO causes a significant reduction in intermolecular chain hydrogen bonding when a specific threshold concentration of DMSO is reached, resulting in a stepwise significant increase in chain mobility. However, when the concentration of DMSO reaches a certain level, the interactions between the DMSO molecules and PMIA chains reach equilibrium, resulting in the polymer chain expansion and mobility becoming stable, and the MSD begins to increase at a slower rate.

In contrast, the MSD and D of PMIA both exhibit an initial increase followed by a decrease as NaCl concentration increases (Figure 11c,d). The D of the PMIA macromolecular chains reaches a peak of 7.4 × 10^3^ Å^2^ ps^−1^ at 10 g L^−1^, then decreases to 1.71 × 10^3^ Å^2^ ps^−1^ at 50 g L^−1^. This behavior can be attributed to the synergistic effect of NaCl and DMSO. At an optimal NaCl concentration, NaCl neutralizes the negative charges on the surface of PMIA fibers, reducing electrostatic repulsion between polymer chains and thereby enhancing the mobility of PMIA molecular chains. However, when the NaCl concentration exceeds a certain threshold, the high ionic strength favors the reformation of hydrogen bonds between fiber molecules, leading to a reduction in the MSD and D. Therefore, in practical dyeing processes, it is crucial to select an optimal NaCl concentration that enhances both the mobility of PMIA molecular chains and the surface charge distribution of the fibers, thereby improving dye adsorption and penetration.

#### 3.2.3 Free Volume Fraction of PMIA

The FFV of the non‐crystalline region of PMIA under different DMSO concentrations is illustrated in Figure 11e. The free volume of PMIA significantly increased from the initial 19.8% to 29%, indicating that the rising DMSO concentration caused the swelling of PMIA. This can also be observed in the corresponding FFV diagram (Figure ^[^ ^11g^ ^]^), where the blue areas representing free volume gradually expand. This indicates that DMSO solubilized the PMIA structure, which resulted in enhanced chain mobility and increased free volume, thus providing more physical space for the diffusion and adsorption of dye molecules and reducing the warping in between dye molecules to the limited adsorption sites, preventing dye aggregation and contributing to uniform dyeing. Therefore, the dyeing results showed that the increase of DMSO resulted in better homogeneity of the dyed PMIA fabrics.

Figure 11f depicts the FFV of PMIA under varying NaCl concentrations. The data reveal that the FFV of PMIA initially rises and then declines as the NaCl concentration increases, corresponding to the trend in the blue free volume within PMIA observed in Figure 11g. The FFV reaches 29.3% and 29.0% at concentrations of 30 g L^−1^ and 40 g L^−1^, respectively. This result is consistent with the findings on hydrogen bonding and MSD. At lower NaCl concentrations, the electrostatic shielding effect, in synergy with DMSO, further promotes the relaxation of molecular chains and the increase in free volume. However, the high concentration of NaCl decreased the chain movement ability of the PMIA amorphous region and reduced the free volume, which affected the diffusion speed of dye molecules into the fiber interior to a certain extent, and it was more difficult for dye molecules to approach the surface of the fiber as the concentration of NaCl increased, so the results of dyeing experiments showed that excessive NaCl decreased the dyeing performance of PMIA, and the homogeneous dyeing deteriorated.

Based on the molecular dynamics simulation results of hydrogen bond counts, mean square displacement (MSD), and fractional free volume (FFV), the molecular mechanism by which DMSO/NaCl induces the rearrangement of PMIA chains lies in the disruption of the internal hydrogen bond network by DMSO, which significantly enhances chain mobility by increasing the fractional free volume. Meanwhile, the electrostatic shielding effect of NaCl reduces the electrostatic repulsion between chain segments, further facilitating the alignment and compactness of molecular chains. During the thermal dyeing process, the chains tend to rearrange into a lower‐energy state, promoting the expansion of crystalline regions. Moreover, the filling effect of dye molecules may further stabilize the alignment of the chains. These findings collectively highlight the critical role of DMSO/NaCl in regulating the crystalline structure of PMIA.

#### 3.2.4 Cohesive Energy of PMIA

**Figure 12:**
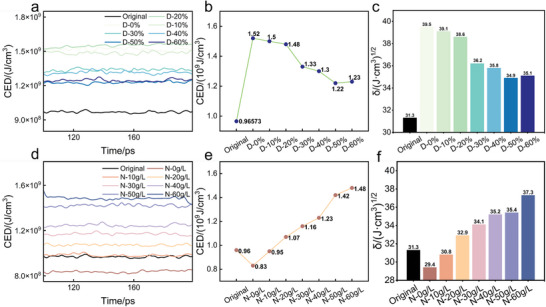
a, d) Change in cohesion energy value. b, e) Mean value of cohesion energy. c, f) Solubility parameters for different models.

CED encompasses van der Waals forces, hydrogen bonding, and other intermolecular interactions, which significantly influence the molecular structure and porosity of PMIA fibers, thereby affecting the diffusion and adsorption of dye molecules. According to the solubility parameter theory, when the difference Δδ = |δ1 – δ2| between the δ of the two substances is small, it indicates that the intermolecular forces between these two substances are matched, and thus exhibit good mutual solubility and compatibility.^[^ ^49^ ^]^ Specifically in this study, when the difference between the modeled δ of PMIA in DMSO/NaCl and the δ of pristine PMIA is small, it implies that that particular ratio of DMSO/NaCl can provide an environment that is more compatible with the molecular structure of PMIA. Therefore, it can be inferred that the DMSO/NaCl solvent mixture has a better swelling effect for PMIA under this condition, which is due to the optimization of the interaction between the solvent and the polymer, which allows the solvent molecules to penetrate into the polymer network more efficiently. From **Figure** **12**b, it can be seen that the CED of PMIA decreased from 1.52 J cm^−3^ at D‐0% to 1.22 J cm^−3^ at D‐50%, which indicates that the increase in the concentration of DMSO led to a decrease in the CED of PMIA, attributed to the disruption of the hydrogen bonding network of PMIA by DMSO/NaCl, which weakened the internal molecular interaction forces within the fiber. In Figure 12e, the CED increases with the rise in NaCl concentration, indicating that the addition of NaCl strengthens the intermolecular interactions, consistent with the above findings that NaCl promotes the increase in hydrogen bonds within the PMIA structure and reduces free volume. The CED results may suggest that NaCl stabilizes and tightens the hydrogen bonds between PMIA fiber molecules, complementing the swelling effect of DMSO. This enables NaCl to partially counterbalance the disruptive effect of DMSO on the fiber structure, thereby maintaining or enhancing the crystallinity and intermolecular forces of fibers.

According to solubility parameter theory, at D‐50% (Figure 12c), the δ value shows the smallest difference from that of the original PMIA, indicating good compatibility and suggesting that the DMSO/NaCl system achieves better swelling of PMIA under these conditions. Figure 12f further reveals that a moderate NaCl concentration in a 50 wt.% DMSO solution maintains good compatibility with PMIA, while an excessive NaCl concentration (60 g L^−1^) results in poor compatibility.

## 4 Conclusion

In conclusion, this study combines experimental and simulation approaches to investigate the dye‐promoting mechanism of DMSO/NaCl on PMIA and explores the structural effects of DMSO/NaCl on PMIA from a microscopic perspective. The dyeing experiment results show that when the DMSO and NaCl concentrations are 50 wt.% and 50 g L^−1^, respectively, the dyed PMIA fabric achieves a good coloration depth (K/S value of 16.0) and dye uptake rate (72.3%) while maintaining excellent mechanical performance. Additionally, colorfastness was significantly enhanced (dry and wet rub fastness at 4–5, wash fastness at 4–5, and lightfastness at 3). The mechanistic insights from the experimental characterization, along with the microstructural analysis from molecular dynamics simulations, indicate that the synergistic effect of DMSO and NaCl surpasses that of either component alone in improving the dyeing performance of PMIA. DMSO disrupts the hydrogen bonding within PMIA, leading to swelling and facilitating dye penetration, while NaCl further enhances dye uptake by modulating the electrolyte environment on this basis. For industrial‐scale applications, DMSO and NaCl offer an effective dyeing system for PMIA, achieving both superior coloration and mechanical performance. Future research should evaluate the performance of various dyes, such as acidic and dispersed dyes, within the DMSO/NaCl system to broaden its applicability. In addition, the combination of experimentation and simulation has great potential for other high‐performance fibers such as para‐aramids and PBO, where structural modification and enhanced dyeability remain key challenges.

## Conflict of Interest

The authors declare no conflict of interest.

## Supporting information



Supporting Information

## Data Availability

Research data are not shared.
