# Supplementary material for: Dimethyl Sulfoxide and Sodium Chloride Modulate the Crystal Structure in PMIA to Enhance Dyeing Performance: Molecular Dynamics Simulation and Experimental Investigations
Source: Adv Sci (Weinh). 2025 Feb 23;12(15):2414544. doi: 10.1002/advs.202414544 (PMC12005731; doi:10.1002/advs.202414544)
Supplement: Supplementary file 1 — Supporting Information [file ADVS-12-2414544-s001.docx]

**Supplementary Material:**

**Dimethyl sulfoxide and sodium chloride modulate the crystal structure in PMIA to enhance dyeing performance: Molecular dynamics simulation and experimental investigations**

Yan Zhuo^a^[[1]](#footnote-1)^#^, Kuang Wang^a#^, Minghui Chen^b^, Zhengke Fan^b^, Zhuangzhuang Sun^a^, Jianli Liu^a^, Yizheng Fu^c^, Aixue Dong^d,e^, Bo Zhu^a*^

^a^College of Textile Science and Engineering, Jiangnan University, Wuxi, 214122, China.

^b^Shaanxi Yuanfeng Textile Technology Research Co., LTD, 710038, China.

^c^School of Materials Science and Engineering, North University of China, Taiyuan, 030051, China.

^d^Key Laboratory of Intelligent Textile and Flexible Interconnection of Zhejiang Province, Zhejiang Sci-Tech University, Hangzhou 310018, Zhejiang, China.

^e^Shaoxing Sub-center of National Engineering Research Center for Fiber-based Composites, Shaoxing University, Shaoxing 312000, Zhejiang, China.

Table S1 Model specific information

| Model | DMSO | Na^+^ | Cl^-^ | lattice parameter/Å |
| --- | --- | --- | --- | --- |
| D-0% | 0 | 10 | 10 | 27.3*27.3*16.2 |
| D-10% | 14 | 10 | 10 | 27.3*27.3*27.6 |
| D-20% | 28 | 10 | 10 | 27.0*27.0*28.6 |
| D-30% | 42 | 10 | 10 | 28.4*28.4*29.1 |
| D-40% | 56 | 10 | 10 | 28.5*28.5*30.5 |
| D-50% | 70 | 10 | 10 | 28.8*28.8*30.9 |
| D-60% | 84 | 10 | 10 | 29.0*29.0*32.0 |

Table S2 Model specific information

| Model | DMSO | Na^+^ | Cl^-^ | lattice parameter/Å |
| --- | --- | --- | --- | --- |
| N-0g/L | 70 | 0 | 0 | 27.7*27.7*31.9 |
| N-10g/L | 70 | 3 | 3 | 27.6*27.6*32.1 |
| N-20g/L | 70 | 6 | 6 | 27.7*27.7*32.5 |
| N-30g/L | 70 | 9 | 9 | 27.9*27.9*33.1 |
| N-40g/L | 70 | 12 | 12 | 28.5*28.5*30.5 |
| N-50g/L | 70 | 15 | 15 | 27.7*27.7*33.2 |
| N-60g/L | 70 | 18 | 18 | 27.7*27.7*33.5 |

There are two criteria to judge whether the system reaches equilibrium: (1) the system temperature reaches equilibrium (temperature fluctuation range ≤15k); (2) the system energy reaches equilibrium (between the range of fluctuation above and below a fixed value)[1], and secondly, it can be judged from the density of the system to determine whether it is consistent with the real structure. Therefore, according to the temperature versus energy curve of the PMIA system in Fig. S1, it can be judged that the PMIA system reaches the equilibrium state. In addition, the density of the PMIA model is 1.27 g/cm^3^, which is not much different from the density of the real amorphous PMIA, 1.33 g/cm^3[2]^, and it can be assumed that the density of the PMIA model at this time is the real density value of the model at the corresponding temperature.


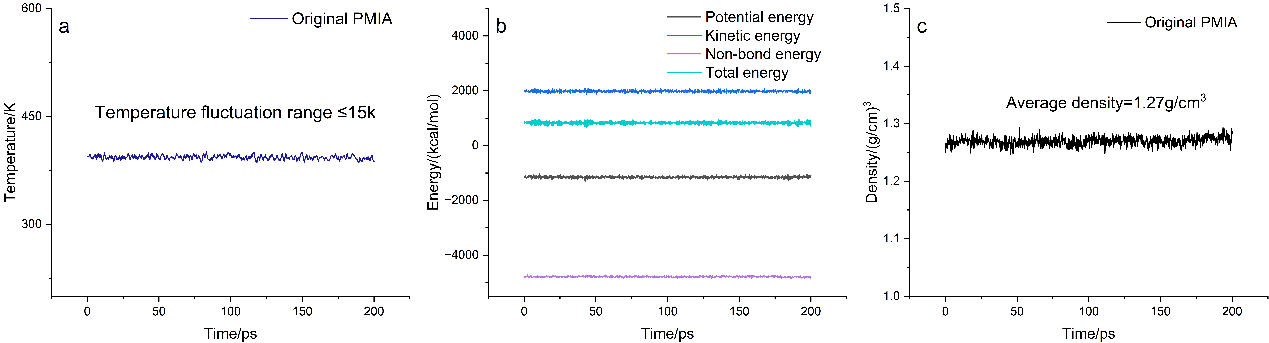


Figure S1 (a) Temperature fluctuation profile of PMIA (b) Energy fluctuation profile of PMIA (c) Density fluctuation profile of PMIA

Hydrogen bonds are weak intermolecular or intramolecular interactions involving electrostatic attraction between a hydrogen atom and a highly electronegative atom (such as N, O, or F). The directionality and saturation of this attraction determine the specific geometric shape and quantity limits of hydrogen bonds, with binding energies typically ranging from 25-40 kJ/mol, stronger than typical van der Waals forces but much weaker than covalent or ionic bonds. The hydrogen bond formula is represented as D-H…A, where D represents the hydrogen bond donor and A represents the hydrogen bond acceptor.


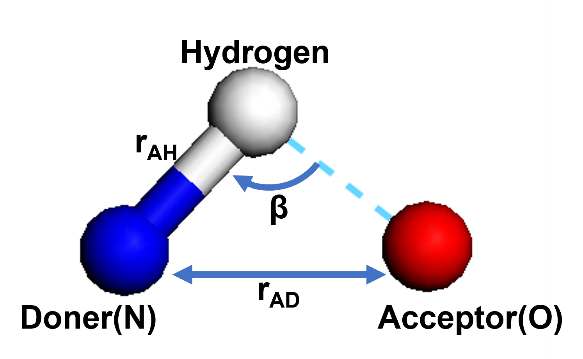


Figure S2 Schematic diagram for the definition of a hydrogen bond.


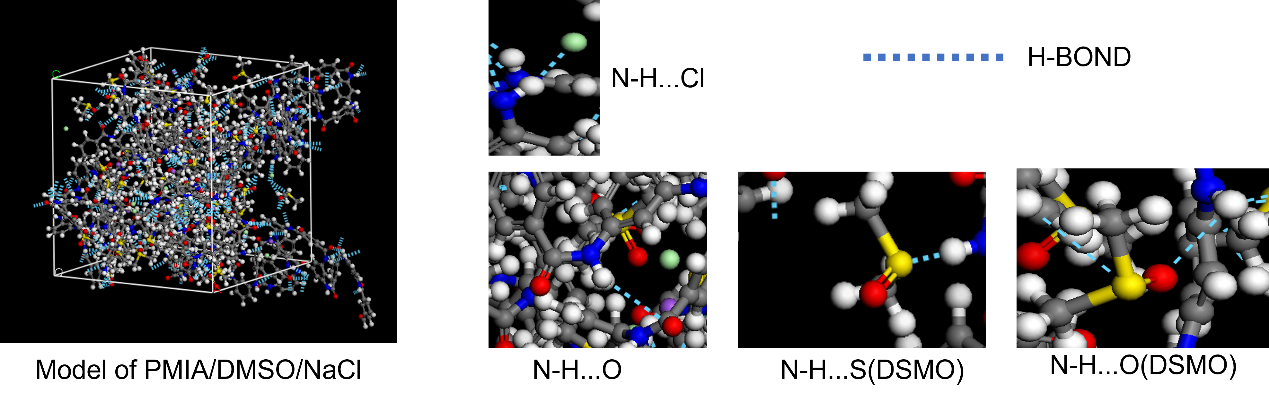


Figure S3 Schematic of hydrogen bonding in the model


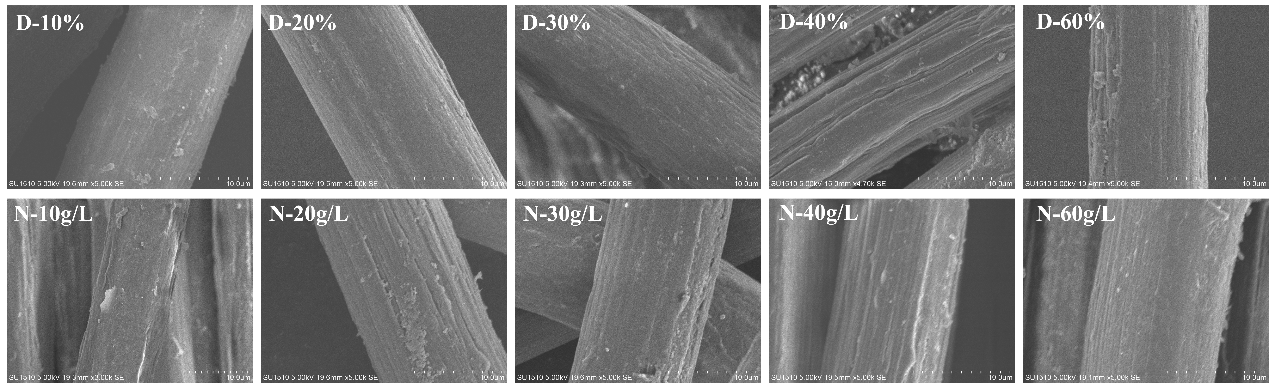


Figure S4 SEM of dyed PMIA


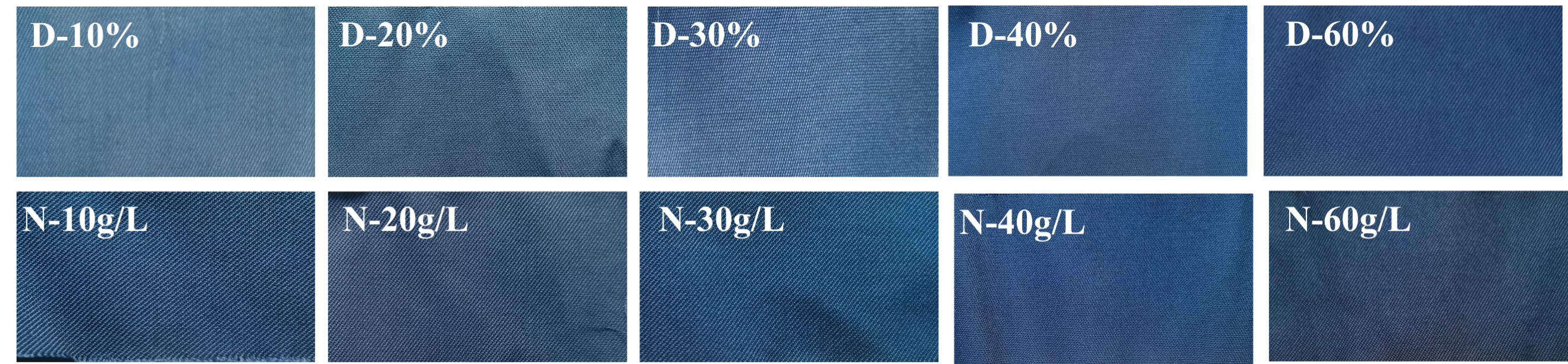


Figure S5 Photographs of dyed PMIA

[1] Fan ZY, Hirvonen P, Pereira LFC, Ervasti MM, Elder KR, Donadio D, Harju A, Ala-Nissila T. Bimodal Grain-Size Scaling of Thermal Transport in Polycrystalline Graphene from Large-Scale Molecular Dynamics Simulations. *Nano Letters*. **2017**, *17*, 5919.

[2] Kazaryan L G TDY, Vasil'ev V A. DAKHIS M A., A TY. Structure of poly-m-phenyleneisophthalamide. *Polymer Science U.S.S.R.* **1975**, *17*, 1797.

[28] Kumar R, Schmidt JR, Skinner JL. Hydrogen bonding definitions and dynamics in liquid water. Journal of Chemical Physics. 2007, 126.DOI: 10.1063/1.2742385

1. # These authors contributed equally to this work. Yan Zhuo, Kuang Wang

   * Corresponding author. E-mail address: zzbb929@163.com (Bo Zhu) [↑](#footnote-ref-1)
